# Quality of care and outcomes in internal medicine patients bedspaced to noninternal medicine units

**DOI:** 10.1097/MD.0000000000025737

**Published:** 2021-05-07

**Authors:** Orly Bogler, Jessica Liu, Ben Cadesky, Chaim M. Bell

**Affiliations:** a Department of Medicine, University of Toronto; b Toronto General Hospital, Division of Internal Medicine, Sinai Health Systems and University Health Network, Toronto; c Department of Medicine, Lakeridge Health, Oshawa; d Department of Medicine, Queen's University, Kingston, Ontario; e Institute for Clinical Evaluative Sciences; f Institute for Health Policy, Management, and Evaluation, University of Toronto; g Department of Medicine, Mount Sinai Hospital, Toronto, Ontario, Canada.

**Keywords:** bed flow, bedspacing, crowding, health systems, quality of care

## Abstract

Hospital overcrowding has led to a practice known as *bedspacing* (in which admitted patients are placed on a different specialty's inpatient ward), yet little is known about the impact of this practice on healthcare quality.

We investigated whether hospital outcome measures differ between bedspaced general internal medicine (GIM) patients vs nonbedspaced patients.

Our retrospective study included patients admitted to GIM wards at 2 academic hospitals (2012–2014), comparing bedspaced to nonbedspaced patients, and identifying adverse events from the hospital's Electronic Patient Record.

We compared these groups with respect to actual length of stay vs the expected length of stay (% ELOS), which is defined as length of stay (LOS) divided by expected length of stay (ELOS), 30-day readmission, adverse events (falls, medication-related incidents, equipment-related incidents, first treatment related incidents, laboratory-related incidents, and operative/invasive events), and in-hospital mortality.

There were 22,519 patients analyzed with 15,985 (71%) discharged from a medical ward and 6534 (29%) discharged from a non-medical ward. Bedspaced patients had shorter lengths of stay (4.1 vs 6.2 days, *P* < .001) and expected lengths of stay (ELOS) (6.1 vs 6.4 days, *P* < .001). Bedspaced patients had a lower percentage of ELOS (% ELOS) than nonbedspaced patients (70% vs 91%, *P* < .001), similar readmission rates (9.8 vs 10.3 events per 100 patients, *P* = .24), lower in-hospital mortality rates (2.6 vs 3.3 events per 100 patients, *P* = .003) and fewer adverse events (0.20 vs 0.60 events per 100 patient days, *P* < .01).

Bedspacing of patients is common. Patients who are bedspaced to off-service wards have better outcomes. This may relate to preferential allocation practices.

## 1 Introduction

Hospital overcrowding remains a pervasive issue.^[1–4]^ At many hospitals, the number of patients requiring admission to specialties such as the general internal medicine (GIM) service exceeds the number of available beds on that specialties’ inpatient ward. As such, patients are frequently admitted to a different service's ward, (e.g., a surgical ward), a process known as *bedspacing.*^[3]^ Typically, a bedspaced patient is geographically separated from the GIM ward and hence, distanced from the physicians caring for them. Depending on the hospital, allied healthcare professionals including nursing, physiotherapy, occupational therapy, social work, and home-care planning are often supplied by the bedspaced ward, so in some cases, varying members of the care team may be unfamiliar with medicine patients.

Recent studies have showed conflicting results in demonstrating differences in quality of care for matched GIM patients who are bedspaced vs nonbedspaced, although those studies were limited by small samples and restrictive methodologies.^[5,6]^ Similar studies of emergency department (ED) “boarders” (in which patients, after admission, temporarily remain in the ED while awaiting an inpatient bed) show that these patients receive poorer quality of care in terms of longer lengths of stay, medication delays, and more adverse events, although other studies are inconsistent.^[7–12]^

We sought to investigate the quality of care of bedspaced patients with respect to length of stay, 30-day readmission, adverse events and mortality. We hypothesized that bedspaced patients would receive poorer quality of care, as compared with nonbedspaced patients.

## 2 Methods

### 2.1 Data collection

All patients admitted to the GIM service at both the Toronto General (TGH) and Toronto Western (TWH) hospitals between April 1, 2012 and December 31, 2014 were included in the study. TGH and TWH are large (>100 medicine beds) tertiary care academic hospitals associated with the University of Toronto and part of the University Health Network. We consolidated the participants in 1 group in order to increase our sample size; these hospitals have similar practices. The GIM service at each hospital is comprised of 4 clinical teams, each led by an attending physician, a senior medical resident, and several first-year residents and medical students. All patients admitted to physicians identified on the GIM physician call schedule at the time of admission were included in the database. Inpatient data were obtained from each hospital's discharge abstraction database. Adverse events were identified from the Electronic Patient Record. The University Health Network Research Ethics Board approval was obtained, and all charts were de-identified.

### 2.2 Patient data and bedspacing

The study included 22,519 patient admissions. We defined bedspaced patients as any admitted GIM patient who was discharged from a non-GIM inpatient ward. All patients who were admitted and discharged without leaving the ED (i.e., “boarders”) were excluded. Patients admitted to a nonmedicine ward (i.e., initially bedspaced) but transferred to a medicine ward and discharged were classified as nonbedspaced patients. We collected the following patient characteristics: age, gender, postal code, admission hospital site discharge diagnosis, admission, and discharge units, discharge destination and comorbidity score. Comorbidity score is a calculated value based on the patient's age, gender, comorbidities, and diagnosis, and ranges from 0 to 4 (4 representing the most medically complex patients) (Table 1).^[13]^

**Table 1:** Baseline characteristics of bedspaced vs nonbedspaced patients (n = 22,519) admitted under the General Internal Medicine service (April 2012–December 2014).

	Bedspaced	Nonbedspaced	*P* value
Patients (n, %)	6534 (29.0)	15985 (71.0)	
Age (mean, standard deviation [SD])	66.8 (19.0)	67.4 (19.0)	.052
Male (%)	3351 (51.3)	8143 (50.9)	.650
Comorbidity Level^∗^			
0	3443 (52.7)	8152 (51.0)	.022
1	1239 (19.0)	2851 (17.8)	.049
2	991 (15.2)	2591 (16.2)	.055
3	627 (9.6)	1756 (11.0)	.002
4	234 (3.6)	635 (4.0)	.179
Discharge destination (%)			
Home	5091 (77.9)	11722 (73.4)	<.001
Died	280 (4.3)	920 (5.8)	<.001
Home with Supports	1904 (29.1)	5268 (33.0)	<.001
Continuing Care Institution	948 (14.5)	2701 (16.9)	<.001
Transfer to another Acute Care Hospital	50 (0.8)	189 (1.2)	.007
Other (None of the above)	165 (2.5)	453 (2.8)	.214

∗ Comorbidity level: Calculated CIHI value based on patient's age, gender, comorbidities, and diagnosis, and ranges from 0 to 4. ^∗∗^*t* test *P* value for age, Chi-Squared test *P* value for all other variables.

### 2.3 Primary outcome

The primary outcome was the percentage of expected length of stay (% ELOS). This value is defined as length of stay (LOS) divided by expected length of stay (ELOS), where LOS is defined as the actual length of stay in hospital (days), and ELOS is a computed estimated value created by the Canadian Institute for Health Information that reflects the expected length of hospitalization when adjusted for age, most responsible diagnosis for hospitalization, medical comorbidities, and in-hospital resource intensity weights specific to Canadian acute care hospitals.^[13]^

### 2.4 Secondary outcomes

The following secondary outcomes were measured: LOS, ELOS, 30-day readmission rate, adverse events (see below), and death. 30-day readmission rate was defined as readmission to either hospital site within 30-days of discharge. Adverse events were defined as a composite of: falls, medication-related incidents, equipment-related incidents, first treatment related incidents, laboratory-related incidents, and operative/invasive events. These adverse events were included if they triggered an incident report.

### 2.5 Statistics

Demographic variables were compared using either a *t* test or Pearson Chi-Squared tests. Generalized Estimating Equations were used to derive averages and 95% confidence intervals for several outcomes (% ELOS, LOS, ELOS, 30-day readmission, death in hospital, and adverse events) for both bedspaced and nonbedspaced patients. The estimates were adjusted for potential confounders (age, gender, comorbidity score, site, and discharge destination [where applicable]) and the confidence intervals account for clustered responses at the level of most responsible diagnosis code. The analysis was performed using R version 3.0.2.

## 3 Results

### 3.1 Baseline

There were 22 519 patients included in the database. Of these patients, 15 985 (71%) were discharged from a medical ward and 6 534 (29%) were discharged from a nonmedical ward (Table 1). Of the bedspaced patients, 51.3% of participants were male as compared to 50.9% of nonbedspaced patients. The average age of bedspaced patients was 66.8 years of age compared to 67.4 years of age in nonbedspaced patients. Comorbidity case mix was similar between groups. Patients in both groups were most frequently discharged to “home” compared to other destinations.

### 3.2 Primary outcome

Bedspaced patients had shorter lengths of stay (4.1 vs 6.2 days, *P* < .001) and shorter expected lengths of stay (6.1 vs 6.4 days, *P* < .001) than nonbedspaced patients. The mean calculated percent ELOS was lower in bedspaced than nonbedspaced patients (70.2% vs 91.7%, *P* < .01) (Table 2).

**Table 2:** Primary and secondary outcomes of bedspaced vs nonbedspaced patients (n = 22,519) admitted under the General Internal Medicine service (April 2012– December 2014) based on discharge location.

	Bedspaced	Nonbedspaced	Estimated difference	*P* value^∗^
LOS (days, 95% CI)	4.1 [3.69,4.58]	6.2 [5.74, 6.71]	2.1 [1.69,2.49]	<.001
ELOS (days, 95% CI)	6.1 [5.78,6.40]	6.4 [6.16, 6.69]	0.3 [0.20,0.47]	<.001
%ELOS (95% CI)	70.2 [62.22,78.18]	91.7 [85.62,97.78]	21.5 [15.91,27.08]	<.001
30-Day-Readmission^†^	9.8 [8.60, 11.24]	10.3 [9.34,11.29]	0.95 [0.87,1.05]	.24
In-hospital Mortality^†^	2.6 [1.55, 4.53]	3.3 [2.03,5.32]	0.80 [0.69,0.93]	.003
Adverse events^‡^	0.20 [0.15,0.26]	0.60 [0.52,0.70]	0.3 [0.42,0.26]	<.001

∗ Wald test *P* values against the null hypothesis of no difference.

† Events per 100 patients, difference given as odds ratio.

‡ Events per 100 patient days, difference given as rate ratio.

### 3.3 Secondary outcomes

There was no difference in 30-day readmission rates between groups (events per 100 patients 9.8 vs 10.3, *P* = .24, OR 0.95, 95% CI 0.87–1.05). Adverse events occurred less frequently if bedspaced, as compared with nonbedspaced patients (events per 100 patient days 0.20 vs 0.60, *P* < .01, RR 0.33, 95% CI 0.26–0.42). Mortality rates were lower in bedspaced patients (events per 100 patients 2.6 vs 3.3, *P* = .003, OR 0.80, 95% CI 0.69–0.93) (Table 2).

## 4 Discussion

This large-scale study of more than 22,000 patients admitted to GIM services at 2 academic centers over a 2-year period found that bedspaced patients had shorter lengths of stay. Bedspaced patients also had significantly reduced adverse events, and in-hospital mortality. Our findings were not in keeping with our hypothesis that bedspaced patients may receive poorer quality of care. We considered whether patients who were sicker were preferentially assigned to the GIM wards and not bedspaced. While we did not have a surrogate for acuity of care, when comparing comorbidity scores between groups, the case mixes for bedspaced vs nonbedspaced patients were similar overall. However, discharge disposition for nonbedspaced patients was more frequently to a destination other than home; that is, complex continuing care or nursing home. That this group was more often discharged to a higher level of care may imply that despite similar comorbidity scores, there may be important differences in groups with respect to care needs.

Moreover, our study found that bedspacing was a common event. In our dataset, almost one third of patients were discharged from a nonmedical ward. This highlights the pervasiveness of bedspacing to ease hospital systems, which may be especially true during periods that have scarcer resources like holidays, weekends, and flu season.

Our results are similar to previous work, which showed that bedspaced patients were not found to receive poorer quality care overall.^[5,6]^ However, our finding with respect to mortality differs from those of Bai et al and Perimal-Lewis et al, who showed that bedspaced patients had higher mortality.^[14,15]^

One of the main strengths of this study was the large size of the population at 2 different centers over a 2-year period. Few studies have attempted to investigate the effect and impact of what is considered a routine practice at many hospitals.

We acknowledge several limitations. First, bedspacing was defined by virtue of discharge location alone, as the majority of our database did not have patient admitting location listed – and patients are often transferred during the course of admission. However, anecdotally, the majority of patient transferring is typically unidirectional – although a nonbedspaced patient could be transferred off the wards, this is typically exceedingly rare. More commonly, patients are “repatriated” to the host GIM wards – and our analysis was not able to account for this practice. However, if anything, repatriation would bias our results to minimize any significant differences between groups. We also took care to exclude emergency room “boarding.” On balance, our definition of bedspacing allowed us to have as close an approximate as possible to a group in which a patient's entire admission was spent bedspaced.

Additionally, though we collected 30-day readmission data from our own hospital sites, we were unable to assess whether patients were re-admitted to other institutions. However, same-hospital readmissions in our jurisdiction accounts for more than 70% of all readmissions.^[16]^ Moreover, we would not expect differential admission practices depending on patient ward location.

Finally, we were unable to assess the type of wards where bedspaced patients were admitted. For example, there may be qualities of various wards (i.e., oncological vs surgical vs stroke services) based on their nursing, allied health, and general familiarity with general internal medicine which may account for differences between the outcomes of bedspaced patients. This would be an area of future investigation.

Contrary to our hypothesis, we did not find that bedspaced patients receive poorer quality of care than nonbedspaced patients. In fact, bedspaced patients fared better on certain quality of care measures, including mortality. One potential explanation may be unmeasured differences between the patient populations. While subtle, there may be an inherent selection bias that is not protocolized or captured by our metrics so that less complex patients, regardless of comorbidity score, are preferentially bedspaced to non-GIM wards. If we had looked at and measured process of care metrics rather than outcomes like mortality, we may have been able to better elucidate key differences within the effects observed. In fact, some of the differences seen in our study compared to previous studies may be a reflection of these differing applications of this practice. Overall, our recommendations would be for hospitals to standardize their approach to bedspacing and attempt to formally derive guidelines for patient selection.

As hospital volumes continue to be an issue, bedspacing will likely remain a strategy to offload busy emergency departments. Reassuringly, our results suggest that this common practice may not result in poorer quality of care. The ultimate goal is to optimize the quality of care of all patients, regardless of geographic location in the hospital so that we can treat the right patient in the right location.

## Author contributions

**Conceptualization:** Orly Bogler, Ben Cadesky, Chaim Belll.

**Data curation:** Ben Cadesky.

**Formal analysis:** Orly Bogler.

**Methodology:** Ben Cadesky.

**Supervision:** Jessica Janine Liu, Chaim Belll.

**Writing – original draft:** Orly Bogler.

**Writing – review & editing:** Jessica Janine Liu, Chaim Belll.
